# Machine-Learning- and Structure-Based Virtual Screening for Selecting Cinnamic Acid Derivatives as *Leishmania major* DHFR-TS Inhibitors

**DOI:** 10.3390/molecules29010179

**Published:** 2023-12-28

**Authors:** Maria Camila Muñoz-Vega, Sofía López-Hernández, Adrián Sierra-Chavarro, Marcus Tullius Scotti, Luciana Scotti, Ericsson Coy-Barrera, Chonny Herrera-Acevedo

**Affiliations:** 1 Department of Chemical Engineering, Universidad ECCI, Bogotá, Distrito Capital 111311, Colombia; maria.munoz.vega@correounivalle.edu.co (M.C.M.-V.); sofia.lopezhe@ecci.edu.co (S.L.-H.); adrianda.sierrach@ecci.edu.co (A.S.-C.); 2 Laboratorio de Investigación en Biocatálisis y Biotransformaciones (LIBB), Grupo de Investigación en Ingeniería de los Procesos Agroalimentarios y Biotecnológicos (GIPAB), Departamento de Química Universidad del Valle, Cali 760042, Colombia; 3 Post-Graduate Program in Natural and Synthetic Bioactive Products, Federal University of Paraíba, João Pessoa 58051-900, PB, Brazil; mtscotti@gmail.com (M.T.S.); luciana.scotti@gmail.com (L.S.); 4 Bioorganic Chemistry Laboratory, Facultad de Ciencias Básicas y Aplicadas, Universidad Militar Nueva Granada, Cajicá 250247, Colombia; ericsson.coy@unimilitar.edu.co

**Keywords:** *Leishmania*, Asteraceae, DHFR-TS, lignans, flavonoids, natural products, machine learning

## Abstract

The critical enzyme dihydrofolate reductase-thymidylate synthase in *Leishmania major* (*Lm*DHFR-TS) serves a dual-purpose role and is essential for DNA synthesis, a cornerstone of the parasite’s reproductive processes. Consequently, the development of inhibitors against *Lm*DHFR-TS is crucial for the creation of novel anti-*Leishmania* chemotherapies. In this study, we employed an in-house database containing 314 secondary metabolites derived from cinnamic acid that occurred in the Asteraceae family. We conducted a combined ligand/structure-based virtual screening to identify potential inhibitors against *Lm*DHFR-TS. Through consensus analysis of both approaches, we identified three compounds, i.e., lithospermic acid (**237**), diarctigenin (**306**), and isolappaol A (**308**), that exhibited a high probability of being inhibitors according to both approaches and were consequently classified as promising hits. Subsequently, we expanded the binding mode examination of these compounds within the active site of the test enzyme through molecular dynamics simulations, revealing a high degree of structural stability and minimal fluctuations in its tertiary structure. The in silico predictions were then validated through in vitro assays to examine the inhibitory capacity of the top-ranked naturally occurring compounds against *Lm*DHFR-TS recombinant protein. The test compounds effectively inhibited the enzyme with IC_50_ values ranging from 6.1 to 10.1 μM. In contrast, other common cinnamic acid derivatives (i.e., flavonoid glycosides) from the Asteraceae family, such as hesperidin, isovitexin 4′-*O*-glucoside, and rutin, exhibited low activity against this target. The selective index (SI) for all tested compounds was determined using *Hs*DHFR with moderate inhibitory effect. Among these hits, lignans **306** and **308** demonstrated the highest selectivity, displaying superior SI values compared to methotrexate, the reference inhibitor of DHFR-TS. Therefore, continued research into the anti-leishmanial potential of these C6C3-hybrid butyrolactone lignans may offer a brighter outlook for combating this neglected tropical disease.

## 1. Introduction

Leishmaniasis is a neglected tropical disease (NTD) caused by protozoan parasites of the genus *Leishmania*, which are transmitted by the bite of infected sandflies. This disease affects millions of people worldwide, particularly in developing countries with poor health infrastructure. The primary clinical forms of the disease are visceral, cutaneous, and mucocutaneous. According to the World Health Organization (WHO), the global burden of leishmaniasis is estimated to be around 700,000 to 1 million new cases each year, with 90% of the cases occurring in just six countries: Afghanistan, Algeria, Brazil, Colombia, Iran, and Syria [1,2]. The sandflies that transmit leishmaniasis are most active at night and breed in wet soil, organic matter, or animal burrows [3]. In Colombia, 10 out of the 20 species that can infect both humans and other living beings are present. The cutaneous leishmaniasis (CL) form is the most frequent (98–99%), with the population under five years old and immunocompromised individuals being the most affected [4,5]. The number of CL cases reported in Colombia in 2022 was 4906, with the departments of Amazonas, Boyacá, Caquetá, Cesar, Córdoba, Cundinamarca, Putumayo, Santander, and Sucre being the most affected areas [6].

Since the late 1980s, *Leishmania*-HIV co-infection has been reported in 35 countries, and there have also been other cases of *Leishmania*-Malaria co-infection, which are associated with the worsening of the clinical condition of patients with leishmaniasis. This co-infection type has increased the disease’s burden due to the greater difficulty of clinical treatment [7,8]. Currently, antimonial compounds are the primary treatment for leishmaniasis; however, they present high toxicity and resistance in some endemic regions. To address these challenges, alternative drugs have been developed, such as liposomal amphotericin B, which significantly reduces the side effects and treatment duration associated with free amphotericin B but is expensive [9,10]. Other drugs, such as paromomycin and miltefosine, have been associated with high toxicity, resistance, and teratogenic and abortive effects, promoting the discovery and development of low-cost, highly effective drugs with low toxicity [11]. Furthermore, it is worth noting that while *Leishmania* is a parasitic disease mainly affecting humans, it also affects animals such as dogs and rodents, which can serve as reservoirs for the parasite and increase the risk of transmission to humans [12,13].

Therefore, efforts to develop effective treatments and control measures must be considered. High-throughput screening (HTS) has been used since the early 1990s to test the activity of large numbers of molecules against different diseases and thereby identify potential hits for drug development [14]. However, the uncertainty of success, as well as the time and screening costs, limit the use of this technique [15]. In recent years, chemoinformatics tools (e.g., molecular docking, machine learning) have been utilized to conduct in silico studies that can predict the interactions between a protein and a ligand, reducing the number of actual laboratory experiments and accelerating the drug discovery process more efficiently and cost-effectively [14,16]. The different research conducted in this field has led to the development of increasingly efficient and better classifying models, which take advantage of large compound databases, opening the possibility of studying diseases that mainly affect poorer populations (NTD), which are not attractive to large industries and big pharma [17].

Leishmaniasis is commonly treated with plants from the Asteraceae family in traditional medicine. Given the diversity of this family (32,913 species) and the wide range of phytochemicals they contain, including alkaloids, coumarins, flavonoids, benzofurans, sterols, and terpenoids, they are considered a promising source of new leishmanicidal compounds [18]. Some secondary metabolites studied in this family have been sesquiterpenoids [19,20], triterpenes [21], phytosterols [22], and kauranes [23]. However, although they have shown activity to inhibit the disease, their pIC_50_ is not large enough, and compounds that are effective at low concentrations and selective against the parasite are preferred. A group of compounds that has not yet been studied, with records reporting promising in vitro activity, is the derivatives of cinnamic acid belonging to the Asteraceae family [24,25,26].

Gouri et al. report some natural inhibitors against *Leishmania* amastigotes, such as luteolin (IC_50_ = 3.12 μM), quercetin (IC_50_ = 10.5 μM), chrysin (IC_50_ = 13 μM), apigenin, myricetin, cinnamic acid (IC_50_ = 0.25 μM), and licochalcone A (IC_50_ = 0.9 μM), which can play an important role in drug discovery [24]. Peixoto et al., on the other hand, evaluated the biological activity of 25 cinnamic acid derivatives against *Leishmania braziliensis* amastigotes, obtaining promising results and finding that aromatic rings with oxygen as a heteroatom had a beneficial effect in terms of activity against *Leishmania* [25]. Considering that heterocyclic compounds have been of great importance for drug development in the pharmaceutical industry, derivatives of cinnamic acid, which is an aromatic carboxylic acid commonly substituted in the *trans* position by an acrylic acid group, represent an interesting starting point for directing studies in the search for possible hits against different species of leishmaniasis [27]. Although some of these compounds have already been studied, many more remain to be analyzed.

Some cinnamic acid derivatives, such as indole-based inhibitors with a Michael acceptor cinnamic ester head, have been tested against human coronaviruses, demonstrating EC_50_ values of 9.14 μM and 10.1 μM [28]. Another area in which their potential has been demonstrated is as antitumor agents. In this context, it has been found that brefeldin A 4-*O*-(4)-dimethylaminocinnamate improves aqueous solubility and exhibits strong cytotoxic activity against HepG2 and BEL-7402 cell lines, with IC_50_ values of 0.29 and 0.84 μM, respectively [29].

Additionally, the compound (*E*)-*N*-(2-(dimethylamino)ethyl)-3-(1H-indol-3-yl)-*N*-(pyridin-2-yl) acrylamide has shown promise as a focal adhesion kinase (FAK) inhibitor for the intervention in metastatic triple-negative breast cancer. It potently inhibits the proliferation, invasion, and migration of TNBC cells in vitro, with an IC_50_ of 8.37 μM [30]. Additionally, these types of compounds have been proven to be potential anti-inflammatory agents by inhibiting Akt/NF-κB and MAPK signaling pathways. Among them, ursodeoxycholic acid–cinnamic acid hybrids showed the best inhibitory activity, with an IC_50_ of 7.70 μM and no significant toxicity [31].

In the present study, a computational approach was undertaken to identify potential inhibitors of the bifunctional enzyme dihydrofolate reductase-thymidylate synthase (DHFR-TS) of *Leishmania major* given its crucial role in the synthesis of DNA in trypanosomatids, which is essential for the parasite’s reproduction [32]. To accomplish this, a custom-made, in-house library containing 314 specialized metabolites derived from cinnamic acid was virtually screened.

Initially, a ligand-based predictive classification model was developed using experimental information on the IC_50_ values retrieved from in vitro assays of reported compounds against *Leishmania*. Simultaneously, employing a hybrid *Lm*DHFR-TS model constructed based on its amino acid sequence [33], a structure-based ranking through molecular docking calculations was performed using the investigated specialized metabolite database. Through a consensus analysis, molecules with the highest probability of being inhibitors by both approaches were classified as possible hits.

These secondary metabolites were further evaluated through in vitro assays using the recombinant *Lm*DHFR-TS, and ADMET properties were calculated to determine their pharmacokinetic properties.

## 2. Results and Discussion

### 2.1. Combined Ligand-/Structure-Based Virtual Screening Approach Using LmDHFR-TS

#### 2.1.1. Ligand-Based Virtual Screening

Initially, a compilation of compounds exhibiting inhibitory activity against *Lm*DHFR-TS was assembled from the ChEMBL database. These compounds underwent classification as either active or inactive, a determination based on their reported IC_50_ values. A cutoff point of pIC_50_ = 5.0 was employed for this classification. The choice of this threshold was grounded in the range of IC_50_ values documented in the ChEMBL database, with an effort to strike a balance between the number of active and inactive compounds. This specific value aims to optimize the representation of chemical space for both active and inactive structure classes while concurrently minimizing the false positive rate of the model.

To refine the dataset, duplicate molecules were eliminated during the data curation process, ensuring the creation of a virtual screening model characterized by high prediction efficiency. Additionally, molecules with an IC_50_ value falling within ±0.1 of the cut-off point were included in the analysis. Ultimately, a total of 790 molecules were chosen for model training. Within this set, 378 were identified as inactive (47.8%), while 412 were recognized as active (52.2%).

In the ligand-based process, VolSurf+ (128) and AlvaDesc (more than 4000) molecular descriptors were calculated from the three-dimensional representation of each compound in the database. For AlvaDesc molecular descriptors, a feature selection was conducted before model training. This process involved removing all constant variables, variables with only one unique value, variables that had at least one sample with a missing value or exhibited autocorrelation greater than 0.95. After this process, 523 molecular descriptors were used for the model construction.

These descriptors were then utilized to construct the random forest (RF) model in Knime software (KNIME 4.5.0, the Konstanz Information Miner, Copyright 2003–2014, www.knime.org (accessed on 2 February 2023)), which comprised 200 decision trees. The Gini index was employed as the split criterion in the RF model to reduce the number of false positive results. The dataset underwent a five-fold cross-validation strategy, where it was divided into five subsets, each containing an 80% modeling set and a 20% validation set. The modeling set was exclusively used for model construction and further subdivided into multiple training and test sets, maintaining an 80%/20% split ratio. These procedures were conducted following the approach described by Fourches et al. [34].

Molecular descriptors play a crucial role in drug discovery and development, serving as representations of the molecular and chemical properties of the compounds under investigation. In this study, the selected descriptors proved to be instrumental. VolSurf+ generates three-dimensional (3D) molecular descriptors based on the distribution of molecular electrostatic potentials and hydrophobicity, encapsulating molecular surface properties, such as size, shape, and electrostatic potential distribution [35,36]. On the other hand, AlvaDesc provides a diverse array of descriptor types, encompassing constitutional descriptors (detailing the number and type of atoms, bonds, and functional groups in the molecule), topological descriptors (representing molecular shape, size, and complexity), electrostatic descriptors (conveying molecular polarity and charge distribution), and quantum mechanical descriptors (pertaining to the electronic structure and properties of the molecule) [37,38].

**Figure 1:**
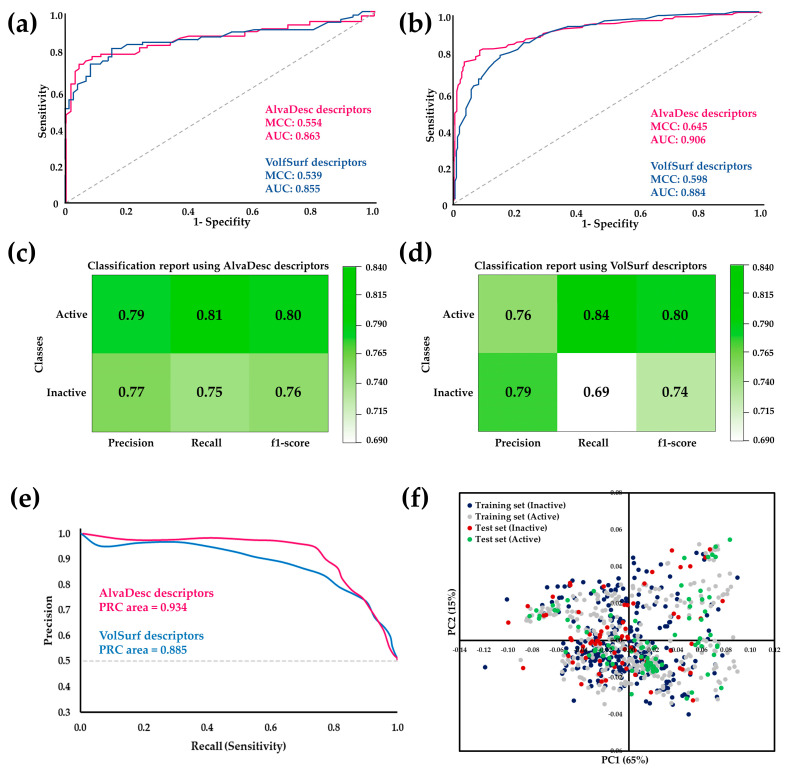
ROC curve comparison for the RF model using AlvaDesc and VolSurf descriptors for (**a**) test sets and (**b**) cross-validation. Performance evaluation of RF using (**c**) AlvaDesc and (**d**)VolSurf descriptors. (**e**) Precision–recall (PR) curves for cross-validation. (**f**) Scatter plots depicting the results of the PCA analysis conducted on the training and test datasets.

The performance of the RF model was assessed to compare the efficacy of the two types of descriptors. This assessment included calculating classification precision, recall, F1-score, and Matthew’s correlation coefficient (MCC). Additionally, receiver operating characteristic (ROC) curves were analyzed, and the area under the ROC curve (AUC) was calculated (Figure 1). These evaluation metrics are commonly utilized to gauge the effectiveness of binary classification models. ROC curves and their AUCs are frequently employed to evaluate the performance of models that generate continuous output scores or probabilities. AUC serves as a scalar measure of the model’s overall ability to distinguish between positive and negative cases [37,39].

According to the parameters presented in Figure 1, it is evident that the MCC and AUC values for both the test sets and cross-validation are higher for AlvaDesc descriptors compared to those obtained for VolSurf descriptors. However, considering that a higher AUC value indicates a more remarkable classification ability of the model and that MCC is expressed in a range of −1 to 1 (where a high value close to 1 suggests a strong correlation between the predicted class and the true class), good values were obtained for both AlvaDesc (AUC: 0.863 and 0.906, MCC: 0.554 and 0.645) and VolSurf (AUC: 0.855 and 0.884, MCC: 0.539 and 0.598) descriptors.

Regarding precision, recall, and F1 score, good and similar values were obtained for both models, except for the recall for inactive compounds in the model created using VolSurf descriptors, which was low, with a value of 0.69. Sensitivity and specificity measures were also calculated to assess the performance of the RF model. For AlvaDesc, the values were 0.807 and 0.752, while for VolSurf, the values were 0.843 and 0.690, respectively. These results indicate a tendency to have few false negatives, a higher value of true negatives, and a lower false positive rate for both descriptors.

The precision–recall (PR) curves, closely related to the ROC curve, were constructed as an evaluation tool for binary classification, enabling the visualization of performance across various thresholds [40]. The results revealed an area under the PR curve of 0.934 for AlvaDesc and 0.885 for VolSurf molecular descriptors, indicating a high-quality model and balanced datasets.

The reliability of the regression model was systematically verified by assessing its applicability domain, ensuring the capability to generate trustworthy predictions. The applicability domain (APD) determination relied on molecular interactions. Results for the training set indicated high reliability rates, reaching 98.1% and 98.4% for the AlvaDesc and VolSurf descriptors, respectively.

Similarly, the test set demonstrated substantial reliability, boasting rates of 96.1% and 100% for the AlvaDesc and VolSurf descriptors, respectively. These results emphasize the model’s dependability in predicting outcomes. In the specific context of cinnamic acid derivatives, the APD calculation yielded a noteworthy 80% of structurally reliable outcomes. This analysis further attests to the model’s robustness in diverse chemical scenarios.

To enhance insights from the APD and visually represent the chemical space distribution, principal component analysis (PCA) was conducted on the datasets employed in this study. This analysis, performed using the training set, projected the results of the test set onto the distribution observed for the training set (Figure 1f). Remarkably, the chemical space of the training set encompassed that of the test set, incorporating molecules classified as both active and inactive.

Regarding the model constructed with AlvaDesc molecular descriptors, those demonstrating greater relevance are those associated with the last eigenvector of the Barysz matrix. This can be achieved either by calculating the average of its coefficients (VE2sign_Dz(p)) or by summing them, with the resulting value weighted by the molecule’s polarizability (VE2sign_Dz(p)) or by Van der Waals volumes (VE2sign_Dz(v)). Additionally, descriptors AVS_B(m) and AVS_B(v) utilize the charge matrix, summing the elements of a specific row or column, and weighting them by mass or Van der Waals volumes, respectively. Furthermore, descriptors based on extended topochemical atom (ETA) indices are considered, specifically those related to hydrogen bond donor atoms (ETA_D_epsiD) [37]. The obtained results regarding the relevance of molecular descriptors are presented in Table S1.

The same analysis, evaluating the relevance of molecular descriptors, was also conducted for VolSurf. The two descriptors with the highest accuracy values were associated with the partition coefficient between 1-octanol and water, namely LgD6 and LgD5, which ranked highest. These descriptors calculate the logarithm of the partition coefficient between 1-octanol and water by summing the logP and the fraction of each species at pH 5 and 6, respectively (Table S1).

Additionally, the LogP n-oct descriptor emerges as one of the most relevant in model construction, along with LdS5, which computes the logarithm of the partition coefficient between 1-octanol and water through a linear equation derived by fitting GRID-derived atom types to experimental data on *n*-octanol/water partition coefficients. Finally, DD1 appears, measuring the difference between the maximum hydrophobic volumes and the hydrophobic volumes of the imported 3D structure calculated at the first level of energy [35,36].

**Figure 2:**
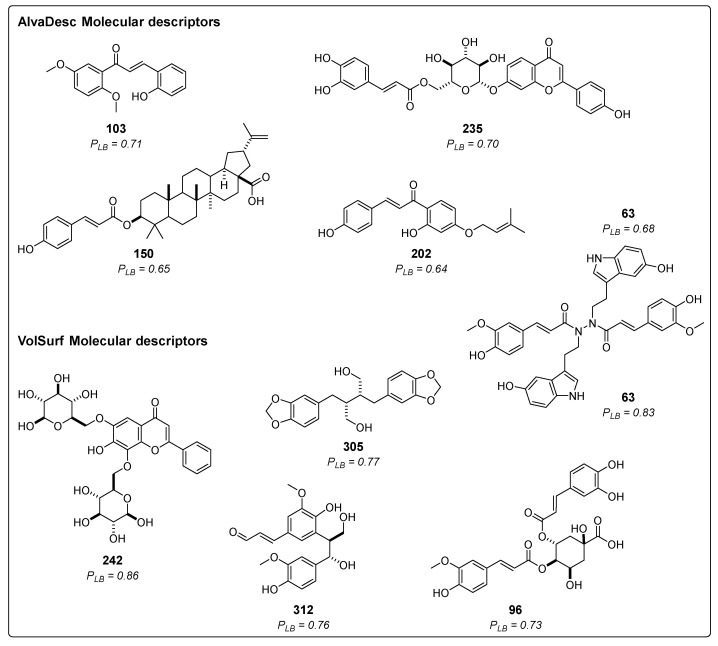
Chemical structures of the five top-ranked cinnamic acid derivatives using a ligand-based virtual screening (LB) with AlvaDesc and VolSurf+ descriptors; *P_LB_* =active probability value.

Ligand-based virtual screening (VS) was utilized to predict the potential inhibitory activity of 314 compounds derived from cinnamic acid in the Asteraceae family, as documented in the literature. Figure 2 showcases the structure and probability of the five best compounds classified using AlvaDesc descriptors. These compounds were (*E*)-2-hydroxy-3′,6′-dimethoxychalcone (**103**) [41], apigenin 7-*O*-(6″-caffeoyl)-glucoside (**235**) [42], montamine (**63**) [43], 3-*O*-*p*-coumaroyl-betulinic acid (**150**) [44], and cordoin (**202**) [45]. Additionally, Figure 2 presents the top five compounds predicted using VolSurf descriptors: 6,8-di-*C*-β-glucopyranosylchrysin (**242**) [46], montamine (**63**) [43], dihydrocubebin (**305**) [47], prebalanophonin (**312**) [48], and 4-*O*-feruloyl 5-*O*-caffeoylquinic acid (**96**) [49].

Among all the tested compounds, 116 were classified as active using AlvaDesc molecular descriptors, with probability values ranging from 0.50 to 0.71. On the other hand, **93** compounds were considered active with VolSurf molecular descriptors, and their probability values ranged from 0.50 to 0.86. Some of these molecules were previously reported to exhibit various activities, such as analgesic activity (**305**), antimalarial activity (**150**), cytotoxic activity (**63**), acting as anticancer agents (**202**), and demonstrating antiproliferative properties (**312**) [43,45,46,47,50,51,52].

Regarding the best compounds, only one contains nitrogen in its structure (**63**). The rest have various oxygen atoms, forming heterocycles or containing carbonyl groups, ethers, and alcohols. Additionally, one of them is a steroid (**150**), and another is glycosylated (**242**).

#### 2.1.2. Structure-Based Virtual Screening

Structure-based virtual screening (VS) was conducted using a hybrid homology model of *Lm*DHFR-TS [33], a bifunctional enzyme with a critical role in the metabolic pathway of *Leishmania* parasites as well as several protozoa species. The *Leishmania* genus is autotrophic for folate and unconjugated pteridines, with the enzyme DHFR-TS playing a pivotal role in the reduction of dihydrofolate to tetrahydrofolate, a cofactor in the biosynthesis of thymine in nucleotide metabolism [53,54].

The *Lm*DHFR-TS hybrid model was constructed in YASARA software v.19.12.14 and subjected to thorough evaluation for reliability and stereochemical qualities through Ramachandran, WHAT IF, and VERIFY 3D analyses. The Ramachandran plot indicated that 96.9% of residues were in favored regions, confirming model satisfaction (Figure S1). VERIFY 3D results, with 92.6% of residues having a reliable 3D-1D score, and WHAT IF evaluation, showing a mean score of −0.594, substantiated the model’s quality. Dihedral assessment revealed optimal values above 1.085, affirming the robustness of the *Lm*DHFR-TS hybrid model [33].

**Figure 3:**
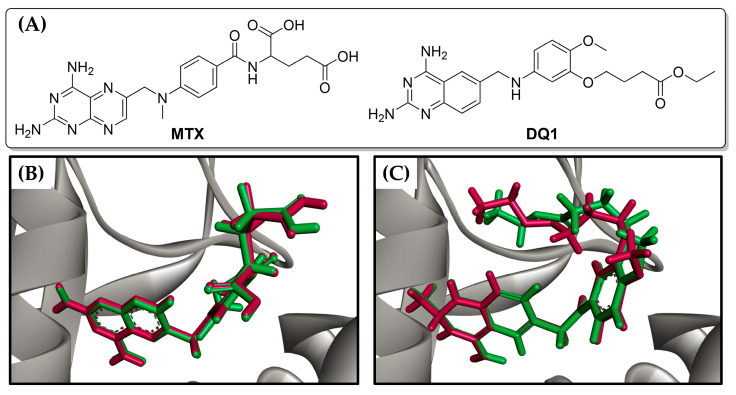
(**A**) Chemical structures of reference ligands: Methotrexate (MTX) and ethyl 4-(5-{[(2,4-diaminoquinazolin-6-yl)methyl]amino}-2-methoxyphenoxy)butanoate (DQ1). Redocking results of (**B**) MTX and (**C**) DQ1 in the active site of *Lm*DHFR-TS. The original ligand conformation is highlighted in red, while the best pose found in the molecular docking procedure is shown in green.

To assess the potential inhibitory capability of cinnamic acid derivatives against *Lm*DHFR-TS, molecular docking calculations were carried out using Molegro software. The results were validated by redocking the co-crystallized ligand, i.e., ethyl 4-(5-{[(2,4-diaminoquinazolin-6-yl)methyl]amino}-2-methoxyphenoxy)butanoate (DQ1), along with the reference inhibitor methotrexate (MTX) (Figure 3).

**Figure 4:**
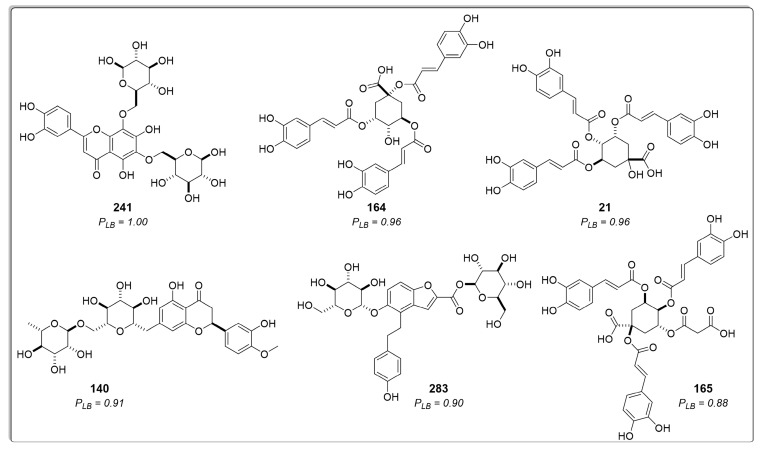
Chemical structure of six of the best-ranked cinnamic acid derivatives that appear as active in the structure-based virtual screening with their respective probability to be active. *P_SB_* = active probability value.

The compounds were ranked based on the predicted docking binding energy using the probability calculation shown below (Equation (1)), as previously reported by Herrera-Acevedo et al. [19,20]. The ten compounds exhibiting the highest probability of being active are presented in Table 1. Ranked compounds that did not previously show high ligand-based probability values but appeared among the best-ranked derivatives through a structure-based approximation are represented in Figure 4 along with their respective structure-based probability (*P_SB_*) values.

(1) $$P_{SB}=(E_{i}/E_{min})>0.5\;\mathrm{and}\;E_{i}<E_{ligand}$$

where $P_{SB}$ is the structure-based probability; $E_{i}$ is the docking energy of compound i, where i ranges from 1 to 314 (cinnamic acid derivatives dataset); $E_{min}$ is the lowest energy value of the dataset; and $E_{ligand}$ is the ligand energy from the co-crystalized inhibitor.

The results showed that the energy-based scoring values were lower for the cinnamic acid derivatives compared to the reference ligands. This suggests that the studied compounds exhibit a higher affinity with the *Lm*DHFR-TS active site in the molecular recognition process. Furthermore, the docking results revealed that 24.5% of the 314 cinnamic acid derivatives dataset had *P_SB_* values above 0.5, and among these top-ranked compounds, 64 had a lower docking score than methotrexate, which achieved −114.15 kJ/mol.

Three of the top-ranked molecules predicted to have high ligand-based probability values based on the RF model also demonstrated high structure-based probability values. Specifically, Compound **242**, ranked fourth in the structure-based classification (Table 1), was the best classified in the ligand-based VS model with VolSurf descriptors. Compounds **235** and **63**, positioned among the top ten compounds in structure-based VS with docking scores of −161.4 kJ/mol and −160.1 kJ/mol, respectively, also showed high ligand-based probabilities. Compound **235** was predicted to be the second-best structure with high potential for inhibition using the model built with AlvaDesc descriptors, while Compound **63** was classified in the top three for both RF models (AlvaDesc and VolSurf molecular descriptors).

**Figure 5:**
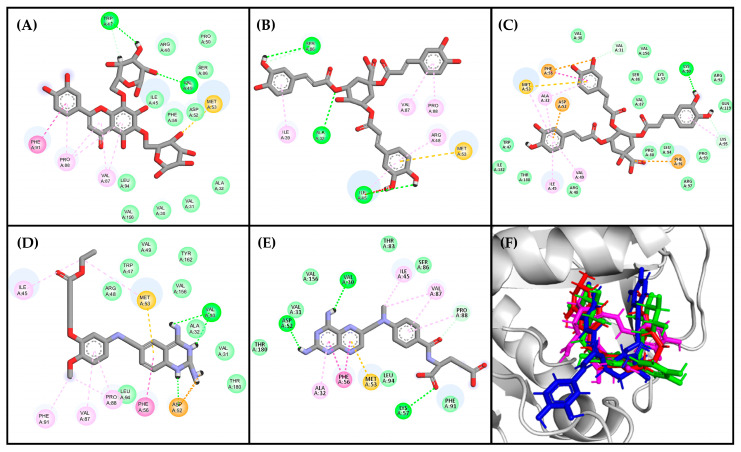
Residual interaction diagrams of (**A**) compound **241**, (**B**) compound **164**, (**C**) compound **21**, (**D**) DQ1, and (**E**) methotrexate. Interacting residues are shown in colored circles and dashed lines depending on the type of interaction: H-bond (lime), van der Waals (green), π–π (purple), π–alkyl (pink), unfavorable (red), carbon H-bond (light green), π–anion (orange), π–sulfide (yellowish orange). (**F**) structural conformations of the coupling between the *Lm*DHFR-TS enzyme and the ligands: DQ1 (red), Compound **241** (green), Compound **164** (pink), Compound **21** (blue).

The analysis of residues for the best poses in the top three compounds revealed that the residues responsible for ligand binding (Val30, Val31, Ala32, Ile45, Trp47, Asp52, Met53, Phe56, Val87, Pro88, Fhe91, Leu94, Val156, Tyr162, and Thr180) have been previously reported in the literature as part of the active site [55]. Certain characteristics of these residues, such as accessibility and charge distribution, enable selective drug design against these protozoans without affecting human enzymes [55]. The interaction diagrams in Figure 5 illustrate that the compound with the highest docking score (Compound **241**, Figure 5C) possesses heterocyclic rings like the reference ligands, with oxygen atoms replacing the nitrogen atoms present in the reference ligands. However, due to the similar electronegativities of nitrogen and oxygen, these atoms favor nearly identical interactions with the enzyme’s active site.

Compounds **164** and **21** lack heterocyclic rings but contain benzene rings, which participate in π–π and π–alkyl interactions. Additionally, these compounds exhibit a relevant number of oxygen-containing groups, such as esters, ethers, and carboxylic acids, facilitating interactions with both residues within the active site and other residues. Specifically, the carboxylic moiety facilitates van der Waals interactions, crucial as they occur with the amino groups in the reference ligands and appear to be important since they are present in the three top-ranked molecules. On the other hand, Compounds **242** and **140**, containing only hydroxyl groups, are less favorable in this binding mode. Although both compounds are isomeric, Compound **164** has few favorable interactions (8 interactions), and Compound **21** has more interactions (25 interactions).

**Figure 6:**
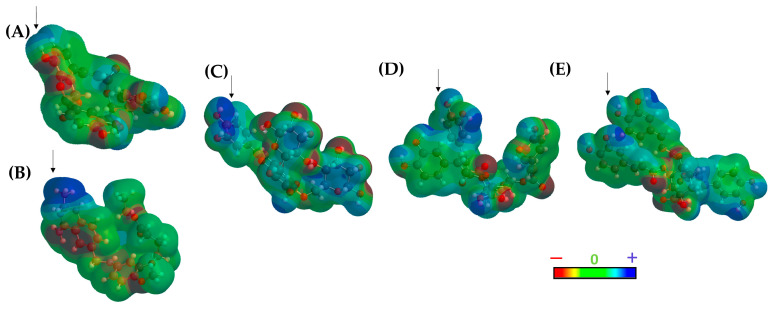
Topological polar surface area (TPSA) map of (**A**) DQ1, (**B**) methotrexate, (**C**) Compound **241**, (**D**) Compound **164**, and (**E**) Compound **21**. The arrows show the electron-deficient region of the molecule.

All ligands adopted a U-shaped conformation like the reference ligands DQ1 and MTX (Figure 5F), and most of them formed robust hydrogen bonding interactions with the enzyme (Val156, Val30, Lys95, Met53, Phe91, and Arg97), which are crucial determinants for binding [53]. To delve deeper into this behavior, a topological polar surface area (TPSA) map was constructed for both the reference ligands and the best-ranked compounds (Figure 6).

The results of the TPSA maps confirmed a similar spatial distribution among the three top-ranked compounds concerning DQ1 and MTX. An electron-deficient region was identified at the top of the molecule (Figure 6, blue area), which is consistently present in all evaluated molecules, including the two reference ligands. This observation rationalized the similar binding behavior within the active site of *Lm*DHFR-TS, particularly with Met53 as a common crucial contact for these test compounds.

The molecular lipophilic potential (MLP) was also analyzed for both ligands and the protein (2). The results obtained from both TPSA and MLP concerning the active site of *Lm*DHFR-TS show that the active site ends are highly polar, explaining the observed charge distribution in cinnamic acid derivatives.

The lipophilic areas of the pocket predominate in the center of the active site, justifying the charge distribution depicted in Figure 6. Additionally, these calculations revealed a pattern of distribution for polar charges for DQ1, MTX, and the three top-ranked structures. However, this was not observed in the lipophilic regions determined in the MLP. The structure **241** exhibits a pattern like MTX, while Ligands **146** and **21** present lipophilic potential like DQ1 (Figure S2).

#### 2.1.3. Consensus Analysis of the Two VS Approaches

A combined approach was employed to determine the potential activity of cinnamic acids against the *Lm*DHFR-TS enzyme and to mitigate the selection of false positive compounds. This approach incorporated probability scores derived from both structure-based and ligand-based virtual screening (VS) methods in conjunction with the true negative rate obtained from the RF model (Equation (2)) [19].

The design of this approach aimed to assign a higher weight to the ligand-based probability scores (considering their reliance on experimental pIC_50_ values), in contrast to the structure-based probability scores, which are founded on protein–ligand interactions. This weighting scheme significantly reduces the risk of incorrectly classifying inactive molecules as active (false positives) [23].

(2) $${CA}_{Lm}=\frac{\left[P_{SB}+\left(\left(1+{TN}_{LB(AD)}\right)\times P_{LB(AD)}+\left(1+{TN}_{LB(VS)}\right)\times P_{LB(VS)}\right)\right]}{\left[3+{TN}_{LB(AD)}+{TN}_{LB(VS)}\right]}$$

where ${CA}_{Lm}$ = combined-approach probability, $P_{SB}$ = structure-based probability, TN = true-negative rate, and $P_{LB}$ = ligand-based probability (AD = AlvaDesc descriptors and VS = VolSurf descriptors).

Table 2 presents the results of the best-ranked compounds calculated from the consensus analysis equation. The compounds ranked among the top five for each method are highlighted in bold. Except for **235**, all compounds were classified as potentially active in all virtual screening approximations used in this study. The consensus analysis identified 110 compounds with combined-approach probability values greater than 0.5; however, only 47% of these compounds (**52**) were classified as active through the three in silico models used in this study (Table S2). Compound **63** (montamine) was the top-ranked compound. Montamine is an indole alkaloid that has been isolated from Asteraceae species, such as *Centaurea schischkinii* and *Centaurea montana*. Previous studies have reported its anticancer properties [43,56], but its efficacy against *Leishmania* has not been investigated.

The second best-ranked compound was 6,8-di-*C*-β-glucopyranosylchrysin (**242**), a derivative of chrysin obtained from *Lychnophora ericoides* (Asteraceae). Compared to Compounds **69** (chrysin) and **231** (techtochrysin), classified as inactive, the glycosylated derivative **242** has more hydroxyl groups, enabling interactions with the enzyme’s active site. In previous studies, chrysin was biofunctionalized with gold particles due to its low bioavailability, poor absorption, and rapid excretion issues, aiming to neutralize *Leishmania* parasites through its activity against the kinase−3 enzyme [57]. However, Compound **242** could represent an alternative due to its hydrophilic character resulting from the glycosyl groups, potentially inhibiting Leishmania parasites by interacting with *Lm*DHFR-TS.

**Figure 7:**
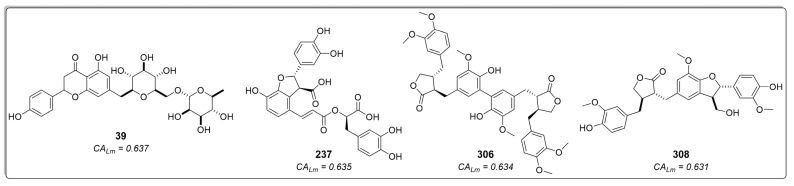
Cinnamic acid derivatives as potential inhibitors of *Lm*DHFR-TS were identified using an approach that combines ligand-based and structure-based virtual screening (VS). *CA_Lm_* represents the combined probability value.

The third- and fourth-best-ranked compounds were 4-*O*-feruloyl-5-*O*-caffeoylquinic acid (**96**) and lucenin-2, 6,8-di-*C*-β-glucopyranosylluteolin (**241**), respectively, both extracted from the genus *Lychnophora*—specifically, *Lychnophora ericoides* [46] and *Lychnophora salicifolia* [49], respectively. Additionally, apigenin 7-*O*-rutinoside (**39**), lithospermic acid (**237**), diarctigenin (**306**), and isolappaol A (**308**)—four cinnamic acid derivatives that previously exhibited moderate values in both RF models and the molecular docking calculations (all classified as active)–appeared among the top ten ranked compounds in the combined approach (Figure 7). Hence, these compounds emerge as interesting antileishmanial candidates, as they exhibit activity across all models and maintain consistency in their probability values. Notably, consensus scoring methods are known to enhance hit rates by diminishing the likelihood of false positives [53,54,55,56,57,58].

The compounds 4-(3,4-dihydroxybenzyl)-2-(3,4-dihydroxyphenyl)tetrahydrofuran-3-carboxy-*O*-β-d-glucopyranoside (**306**) and 7-(3,4-dihydroxyphenyl)-3′,4′-dihydroxy-7,8,7′,8′-tetrahydronaphtho [8,8′-c]furan-1(3*H*)-one (**308**) are two lignans found in certain species of Asteraceae. Notably, *Hypochaeris radicata* (native to Europe, northern Asia, and parts of North Africa) and *Arctium lappa* (native to Europe and Asia) have been reported as natural sources of these compounds. However, *A. lappa* is widely disseminated in America, and *H. radicata* has also become invasive in regions as far-flung as New Zealand and Chile [59]. Conversely, compound **237**, lithospermic acid, is a common polycyclic phenolic carboxylic acid that has been isolated from species of multiple botanical families, including Lamiaceae and Asteraceae. It has demonstrated a wide range of beneficial properties, acting against cardiovascular diseases and hepatitis. It allows endothelium-dependent vasodilatation, lowers blood pressure, and produces antioxidant effects [60,61].

### 2.2. Molecular Dynamics Simulations

Conducting molecular dynamics (MD) studies aimed at evaluating protein–ligand stabilities involved considering various factors such as solvent, ions, pressure, and temperature for Compounds **237**, **306**, and **308**. These three compounds emerged as potential inhibitors of *Lm*DHFR-TS based on the consensus analysis of the methodologies employed in this study. Methotrexate (MTX) served as the reference ligand.

The assessment of structural stability was accomplished through root mean square deviation (RMSD) measurements. Over the simulated period of 100 ns, all tested compounds exhibited comparable behavior in relation to the apoenzyme of *Lm*DHFR-TS (apo*Lm*DHFR-TS, the protein without a ligand) and the *Lm*DHFR-TS···MTX complex.

**Figure 8:**
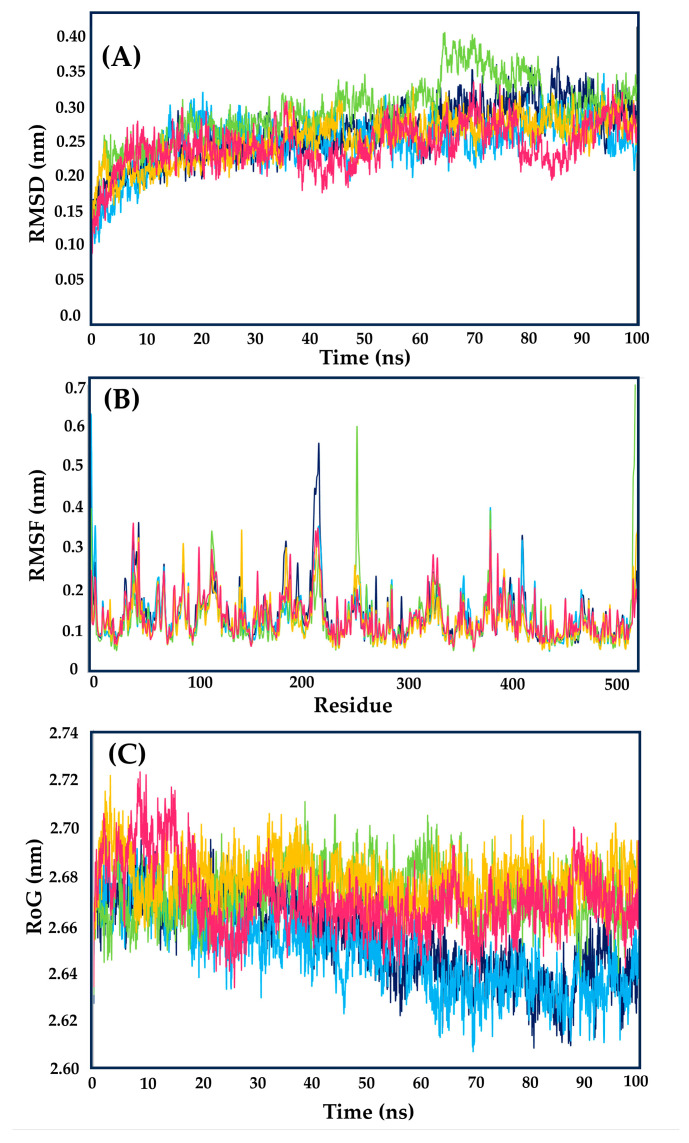
(**A**) Root mean square deviation (RMSD), (**B**) root mean square fluctuation (RMSF), and (**C**) radius of gyration (RoG) values within the LmDHFR-TS binding site obtained after molecular dynamics simulations. Apoenzyme (blue); DHFR-TS···MTX complex (cyan); DHFR-TS···**237** complex (light green); DHFR-TS···**306** complex (yellow) and DHFR-TS···**308** complex (pink).

Upon detailed examination of Figure 8A, it becomes evident that during the initial 30 ns of the simulation, the complexes formed by *Lm*DHFR-TS with the three analyzed ligands exhibit behavior like that of the complex with MTX and apo*Lm*DHFR-TS. However, after the 40 ns mark, derivatives **237** and **308** display a higher level of disturbance, with RMSD values fluctuating between 0.10 and 0.15 nm (Figure 8A).

Structure **306**, in contrast, maintains behavior like the *Lm*DHFR-TS···MTX complex throughout the entire 100 ns simulation, with a minor RMSD variation (close to 0.10 nm) compared to the other two analyzed derivatives. This suggests favorable stability of the protein, as the apo*Lm*DHFR-TS experiences a variation of 0.15 nm, with a minimum observed at 40 ns and an increase in RMSD values reaching a maximum near 85 ns of the simulation.

Concerning RMSF values (Figure 8B), all examined compounds displayed similar behavior, although specific cases revealed distinct characteristics. Residues Glu218 and Thr410, situated in the protein’s loop regions, exhibited the highest fluctuations for the apoenzyme, with Glu218 showing approximately twice the RMSF value compared to the complexes with MTX and the tested cinnamic acid derivatives.

Among the selected compounds, Compound **237** demonstrated higher fluctuations in the loop regions than the other derivatives and MTX, with Gly118, Arg254, and Arg380 being the most variable amino acids. Compounds **306** and **308** exhibited a similar behavior throughout the simulation, showcasing reduced flexibility when complexed.

The critical amino acid residues involved in binding to *Lm*DHFR-TS· exhibited relatively stable behavior, with RMSF values ranging from 0.10 to 0.20 nm throughout the simulation. Among these residues, Phe91 and Lys95 demonstrated higher variation, exceeding 0.20 nm. In contrast, Arg97 and Val156 exhibited minimal fluctuation, with values close to 0.10 nm. Notably, Val156 in apo*Lm*DHFR-TS and the MTX complex displayed lower fluctuation (approximately 30%) compared to the three analyzed cinnamic acid derivatives.

Conversely, Arg97 displayed values between 0.09 and 0.12 nm. Structure **306** achieved a remarkable value of 0.09 nm, even lower than observed for the MTX complex, while Structures **237** and **208** showed values like those of the apoprotein. Throughout the simulation, the *Lm*DHFR-TS complex with Structure **306** consistently promoted protein stability, evidenced by lower RMSF values in this complex, except for residues Leu145 and Lys90.

The structural compactness and mobility of the protein–ligand complexes were assessed throughout the simulation using the radius of gyration (RoG) plot (Figure 8C) [23]. In the initial half of the 50 ns simulation, complexes with cinnamic acid derivatives displayed RoG values indistinguishable from those of the control MTX and apo*Lm*DHFR-TS, ranging from 2.64 nm to 2.70 nm.

This indicates a high level of stability and low fluctuations in the tertiary structure. However, after 60 ns, Compounds **237**, **306**, and **308** exhibited similar behavior (varying between 2.64 nm and 2.70 nm) with increased perturbation compared to the DHFR-TS···MTX complex and the apoenzyme, maintaining a consistent mean value with fluctuations ranging from 2.62 to 2.64 nm.

Following molecular dynamic simulations, binding free energies for complexes involving Compounds **237**, **306**, **308**, and the control (MTX) with *Lm*DHFR-TS were determined using the MM/PBSA method. The complexes of benzylbutyrolactone-type lignans (**306** and **308**) and the polyphenolic acid (compound **237**) with *Lm*DHFR-TS showed binding free energies of −111.1 kJ/mol, −81.0 kJ/mol, and −91.6 kJ/mol, respectively. In all cases, the energy was higher than the −124.5 kJ/mol observed for the complex of MTX with *Lm*DHFR-TS (Table 3).

All complexes under evaluation, including the MTX reference, exhibited a consistent contribution pattern characterized by negative energy values arising from van der Waals, electrostatic, and solvent-accessible surface area (SASA) parameters influencing the binding free energy. The van der Waals parameter, displaying the most substantial negative contribution, registered values lower than −209 kJ/mol. This finding implies that non-polar electrostatic interactions play a pivotal role in the molecular recognition of the LmDHFR-TS binding site by the tested compounds.

Concerning polar solvation, all compounds made positive contributions to the total binding energy, with similar values observed for Compounds **237**, **308**, and MTX. Conversely, diarctigenin (**306**) exhibited a lesser contribution to this parameter. Additionally, electrostatic interactions negatively influenced the binding free energies, with MTX showing a more significant negative contribution of −57.3 kJ/mol. Meanwhile, the impact of electrostatic interactions for the evaluated cinnamic acid derivatives ranged from 35% to 50% relative to the reference ligand.

### 2.3. In Vitro Enzymatic Activity Inhibition for Selected Cinnamic Acid Derivatives (Compounds ***237***, ***306***, and ***308***) against LmDHFR-TS and HsDHFR

To validate the outcomes of our combined approach utilizing two virtual screening (VS) methodologies, we conducted in vitro enzymatic inhibition assays on five compounds sourced from our in-house library. Compounds **237**, **306**, and **308**, identified as active in all approaches, were selected, along with hesperidin (**140**), a notable flavonoid recognized for its reported antileishmanial activity through apoptosis induction and sterol C-24 reductase inhibition [62]. Isovitexin 4′-*O*-glucoside and rutin, demonstrating moderate levels of activity and categorized as inactive in one of the three approaches, were also assessed against *Lm*DHFR-TS, with methotrexate serving as the positive control.

The determination of IC_50_ values involved analyzing concentration-response curves within the 0.1–128 μM range, employing spectrophotometric monitoring of enzymatic activity in a standard DHFR assay. This investigation yielded a spectrum of values ranging from 6.1 to 53.2 μM, corresponding to pIC_50_ values between 4.27 and 5.21. Notably, Compounds **237**, **306**, and **308** demonstrated the highest activity against *Lm*DHFR-TS. Hesperidin (IC_50_ = 21.6 μM) exhibited substantial activity against the target among the three evaluated flavonoids, with IC_50_ values of 53.2 μM and 41.7 μM for isovitexin 4′-*O*-glucoside and rutin, respectively (Table 4).

Structurally, we sought to establish a correlation between the inhibitory activity against LmDHFR-TS and the interaction of hydrogen bond acceptors and donors, particularly carbonyl and hydroxyl groups. Among the lignans—**306** and **308**—the presence of the γ-butyrolactone moiety highlighted that the most active compound (**306**) possessed a higher number of carbonyl groups compared to **308**—a feature shared with lithospermic acid (**237**). However, the glycosylated flavonoids (hesperidin, isovitexin 4′-O-glucoside, and rutin) exhibited low inhibitory activities, suggesting that the abundant hydroxyl groups may negatively impact inhibitory activity.

Following this, we calculated the selectivity index (SI) based on the results obtained from in vitro tests using the recombinant protein of Homo sapiens (Hs) DHFR. The IC_50_ values against HsDHFR revealed a distinct pattern, implying different mechanisms of action for these two proteins. Moderate SI values were observed, with both benzylbutyrolactone-type lignans (Compounds **306** and **308**) exhibiting the highest SI values—4.6 and 4.4, respectively. Notably, both lignans demonstrated higher SI values than MTX, employed as a positive control (Table 4)

### 2.4. Pharmacokinetic Properties Predictions

The pharmacokinetic properties, encompassing absorption, distribution, metabolism, excretion, and toxicity (ADMET), of Compounds **237**, **306**, and **308** were predicted using ADMETlab 2.0 and OSIRIS DataWarrior 5.5.0 [63,64]. Multiple approaches were employed to evaluate oral bioavailability, yielding mixed results. While all compounds adhered to Lipinski’s “rule of five” [65], none met the criteria set by Pfizer [66] and GSK [67], suggesting potential challenges in oral bioavailability (Table S3).

Regarding cytochrome P450 (CYP) and its isoenzymes, compound **237** exhibited a significant probability of inhibiting CYP2C9. Similarly, Compounds **306** and **308** demonstrated potential inhibition of CYP2C19, CYP2C9, and CYP3A4, indicating potential impacts on the metabolism of other drugs. Conversely, Compound **237** was predicted to act as a substrate for CYP2C9, while Compounds **306** and **308** were associated with CYP1A2, CYP2C19, CYP2C9, CYP2D6, and CYP3A4, suggesting that they could be metabolized by these isoenzymes. Furthermore, none of the studied compounds exhibited mutagenic, tumorigenic, reproductive, or irritant effects. Identifying potential hERG channel blockers is crucial for assessing the risk of cardiotoxicity [68], and for the three structures, the probabilities of hERG blocking were at most 0.212.

## 3. Materials and Methods

### 3.1. Cinnamic Acid Derivatives In-House Dataset

A custom-made, in-house virtual library of 314 distinct cinnamic acid derivatives was built from 76 scientific articles using various search criteria, including keywords such as Asteraceae, Cinnamic Acid Derivatives, Lignans, Polyphenols, Flavonoids, and others. ChemAxon MarvinSketch (ChemAxon, version 21.18.0 (2021), a calculation module developed by ChemAxon, https://www.chemaxon.com/, accessed on 12 January 2023) was used to design all the structures.

The three-dimensional (3D) structures for the entire set were generated using Standardizer software (JChem, version 21.18.0 (2021), a calculation module developed by ChemAxon, https://www.chemaxon.com/, accessed on 12 January 2023). This software standardized the structures, added hydrogens, performed aromatic form conversions, and refined molecular graphs in three dimensions. The process employs a divide-and-conquer strategy, wherein the structure is partitioned into smaller fragments. These fragments are then organized into a tree based on connectivity information. Conformers generated for the initial structure, represented by the root node in the tree, undergo optimization. The tree-building process incorporates a proprietary extended version of the Dreiding force field [69]. The final dataset was saved in special data file (.sdf) format.

### 3.2. Classificatory Machine Learning Models

The analyses described below utilized Knime 4.5.0 software (KNIME 4.5.0, the Konstanz Information Miner, Copyright 2003–2014, www.knime.org (accessed on 2 February 2023)) [70]. The process commenced with importing those descriptors generated by the Volsurf+ [35,36] and AlvaDesc [37,38] programs in CSV format.

Subsequently, these descriptors underwent segmentation via the “Partitioning” node, implementing the stratified sampling option, with 80% of the initial dataset designated as the training set and the remaining 20% composing the test set. Random splits were also explored while maintaining consistent ratios for both training and test sets.

The model’s creation processes entailed utilizing the modeling set and the RF algorithm, executed through a five-fold cross-validation procedure employing WEKA nodes. This approach provides a robust and efficient means to evaluate a model’s performance by partitioning the data into five subsets for testing and training, facilitating model selection and generalization assessment [23].

The applicability domain was assessed through Euclidean distances, targeting compounds in the test set with potentially unreliable predictions. A compound was considered unreliable if its applicability domain value exceeded d + Zσ, where d represents the average Euclidean distance, and σ is the standard deviation of the samples in the training set. These samples exhibited Euclidean distance values lower than the average when compared to all training set samples, with Z serving as an empirical cutoff value set at 0.5 by default [20,71].

To complement these findings and provide a more comprehensive visualization of the chemical space within the datasets used for model construction, principal component analysis was conducted on the four datasets, encompassing both active and inactive structures for both the training and test sets. This analysis was executed using Unscrambler X (The Unscrambler^®^ X v10.3 User Manual Version 1.0 CAMO SOFTWARE AS, Oslo, Norway).

The RF models were fine-tuned with 200 trees and a random number generator seed of 1, and the Gini index was utilized as the split criterion for both the training and cross-validation sets. These parameter choices were informed by a thorough evaluation of relevant hyperparameters for the machine learning model. The “number of trees” parameter was explored across a range from 100 to 1000, with 200 trees identified as the optimal selection for achieving the best quality parameters. Subsequently, the Gini index was meticulously chosen as the preferred split criterion (Table S4).

Performance analysis of the selected models encompassed an evaluation of both internal and external aspects, incorporating parameters such as sensitivity (true-positive rate), specificity (true-negative rate), and accuracy (overall predictability), derived from the confusion matrix. To offer a more comprehensive understanding of the model’s performance beyond accuracy, the ROC curve was employed. Generated through an “ROC curve” node, this curve relies on sensitivity and specificity. The AUC values derived from the ROC curve range from 0.5, indicating an inability to distinguish between the two groups, to 1, signifying perfect separation without overlap [72]. Additionally, the Matthews correlation coefficient (MCC) was calculated, in which a value of 1 represents perfect prediction, 0 denotes random prediction, and -1 indicates complete disagreement between prediction and observation [73].

Moreover, a performance evaluation of the RF model using AlvaDesc and VolSurf+ descriptors was conducted. This evaluation included precision, recall, and F1 score metrics for both active and inactive sets.

### 3.3. Molecular Docking Calculations

Molecular docking calculations involved the hybrid model of *Lm*DHFR-TS bound to methotrexate (MTX) [33] and the three-dimensional structures of the cinnamic acid derivatives. We conducted these calculations using Molegro 6.0.1 software.

To ensure consistency, we removed all water molecules from both the enzyme and compound structures, and we prepared them to use the software’s default settings. The MolDock scoring function was utilized, considering internal ES, internal H-bond, and Sp2–Sp2 torsions as criteria for evaluating the ligands.

The molecular docking process was executed through 10 runs utilizing the MolDock SE algorithm. It allowed for a maximum of 1500 interactions, maintained a population size of 50, included up to 300 steps, employed a neighbor distance factor of 1.00, and returned a maximum of 5 poses. To cover the enzyme’s ligand-binding site, we established a grid with a 15 Å radius and 0.30 Å resolution [23,33].

Our results were categorized according to docking scores, reflecting the free energy or affinity of the interactions. Each calculation was repeated three times to ensure reliability. For comparison, we employed methotrexate (MTX) as a control.

Topological polar surface area (TPSA) maps were calculated using Spartan 14 for Windows Spartan’14 (Wavefunction Inc., Irvine, CA, USA) [74]. Molecular lipophilic potential (MLP) maps for ligands were calculated in Molinspiration (Molinspiration, Cheminformatics free web services, https://www.molinspiration.com (accessed on 24 November 2023), Slovensky Grob, Slovakia). For *Lm*DHFR-TS, MLP and TPSA were calculated using ChimeraX [75]. The visualization of two-dimensional residual interaction diagrams was accomplished using Discovery Studio Visualizer v21.1.0.20298 (BIOVIA, Dassault Systèmes, San Diego, CA, USA) [23,33].

### 3.4. Molecular Dynamics Simulations

Molecular dynamics (MD) simulations were conducted in YASARA Structure v. 19.12.14 [76], employing the AMBER14 force field to model the enzyme and ligand–enzyme systems. Before the simulations, each protein underwent hydrogen bond optimization, and chloride (Cl^−^) and (Na^+^) ions were added to the model systems through the transferable intermolecular potential 3-point (TIP3P) employing 0.997 g/L density for solvating the simulation cell. Acid dissociation constant values (pKa) were calculated for enzymes’ titratable amino acids with the *H*-bonding network and the side-chain placement using a rotamer library (SCWRL) algorithm. Periodic boundary conditions were applied to facilitate the simulations, involving a cell size set 10 Å larger than the protein’s size in all instances.

An initial 5000-cycle energy minimization step was carried out using the steepest gradient approach. MD simulations used the particle-mesh Ewald (PME) method to account for long-range electrostatic interactions (8-Å cut-off distance). The simulations were performed under physiological conditions at 298 °K, pH 7.4, and 0.9% NaCl. Temperature control was maintained using a Berendsen thermostat while keeping the pressure constant. A multiple-time step algorithm with a time step of 2.00 fs was employed. Finally, MD simulations were run for 100 ns under constant pressure, and the Berendsen thermostat, with snapshots saved at intervals of 100 ps, used the YASARA macro (md_run.mcr) for all simulation phases. Subsequent analyses were also carried out using the default YASARA macro scripts. The molecular mechanics Poisson–Boltzmann surface area (MM-PBSA) method was employed to calculate the binding free energies of apoenzyme and enzyme–ligand complexes from the resulting MD trajectories using the g_mmpbsa tool in Gromacs 5.0.5 (open source, http://www.gromacs.org (accessed on 17 May 2023)) [77] on an Ubuntu 12.04 server, using NPT and periodic boundary conditions, as previously reported [33,78].

### 3.5. LmDHFR-TS and HsDHFR Enzymatic Inhibition Assays

Purification and kinetic characterization of the recombinant *Lm*DHFR-TS protein were performed according to the previously reported procedures [33,79,80], while *Hs*DHFR protein was obtained from the commercial assay kit (CS0340, Merck KGaA, Darmstadt, Germany). Thus, the in vitro evaluation of the top-ranked selected compounds (**237**, **306**, **308**, hesperidin, rutin, and isovitexin 4′-*O*-glucoside) for inhibitory activity against *Lm*DHFR-TS and *Hs*DHFR was conducted using a spectrophotometric assay under standard DHFR conditions. These tested compounds were available from our in-house compound library. Rutin, lithospermic acid, and rutin were commercially purchased (>98%, Merck KGaA, Darmstadt, Germany). Isolappaol and diarctigenin were isolated from a commercial *A. lappa* powdered root extract (Prescribed For Life, Fredericksburg, TX, USA) through successive column chromatography, whose spectroscopic data was identical to those of previous reports [81,82].

The assay was conducted with either *Lm*DHFR-TS or *Hs*DHFR (2.7 nM), bovine serum albumin (BSA, 1 mg/mL), *N*-[tris(hydroxymethyl)-methyl]-2-aminoethanesulfonic acid (TES) buffer (100 mM, pH 7.0, 150 mM β-mercaptoethanol, 2 mM ethylenediaminetetraacetic acid (EDTA)), and nicotinamide adenine dinucleotide phosphate (NADPH, 100 μM), along with varying concentrations of the test compounds (0.1–128 μM). The reaction was initiated by adding the substrate (7,8-dihydrofolate (H2F), 20 μM) and monitored for 360 s at 340 nm, measuring the oxidation of NADPH to NADP+. This allowed the determination of the initial reaction rate (Vo) through linear regression analysis of the resulting absorbance profile.

All measurements were conducted in triplicate, and methotrexate (MTX) served as a positive control [33]. The resulting Vo values were utilized to calculate the % inhibition, expressed as 100 − (*Ri*/*Rc* × 100), where *Ri* is the Vo in the presence of the inhibitor, and *Rc* is the Vo in the absence of inhibitors (1% DMSO *v*/*v* final concentration). % inhibition was measured for at least five concentrations (0.1–128 μM) for each test compound (cinnamic acid derivatives and MTX), and concentration-response curves (% inhibition vs. Log[inhibitor]) were constructed using non-linear regression in GraphPad Prism 7.0 (GraphPad, San Diego, CA, USA). [33].

### 3.6. Pharmacokinetic Properties Predictions

The ADMET parameters for Compounds **237**, **306**, and **308** were calculated using ADMETlab 2.0, an integrated online platform for predicting ADMET properties [63]. Additionally, drug toxicity predictions were conducted using OSIRIS DataWarrior v.5.2.1, considering parameters such as mutagenicity, tumorigenicity, reproductive effects, and irritability [64].

## 4. Conclusions

This study identified three cinnamic acid derivatives, lithospermic acid (**237**), diarctigenin (**306**), and isolappaol A (**308**), as potential inhibitors of *Lm*DHFR-TS using a combined virtual screening approach (structure/ligand-based). Two random forest models were built using different molecular descriptors. Sensitivity and specificity measures were obtained to evaluate the RF model’s performance. The models classified 116 (AlvaDesc) and 93 compounds (VolSurf) as active, showing a tendency to minimize false negatives.

Molecular docking revealed that 24.5% of the 314 cinnamic acid derivatives had values above 0.5, with 64 of them having a lower docking score than methotrexate, the reference ligand. A consensus analysis combining the RF models with molecular docking identified 110 compounds with combined-approach probability values greater than 0.5. From them, 47% were classified as active through the in silico models, identifying some compounds with potential leishmanicidal activity that a single approach had not previously highlighted. Lithospermic acid (**237**), diarctigenin (**306**), and isolappaol A (**308**) were among the top-ranked compounds, and their binding mode was evaluated using molecular dynamics. Finally, in vitro assays using recombinant *Lm*DHFR-TS validated the computational results, with **237**, **306**, and **308** exhibiting significant activity against *Lm*DHFR-TS. However, moderate selective indices (SIs) were observed when assays were performed using *Hs*DHFR. Despite this finding, higher SI values than MTX were observed. Thus, these three tested compounds emerged as an interesting alternative as hits against *Lm*DHFR-TS; however, specific assays against the parasitic forms of *Leishmania major* are required to extend a clearer prospect for fighting this neglected tropical disease.

## Figures and Tables

**Table 1:** Chemical structure of six of the best-ranked cinnamic acid derivatives that appear as active in the structure-based virtual screening with their respective probability to be active. *P_SB_* = active probability value.

Rank	Ligand	Docking Score (kJ/mol)	SD	RMSD
1	**241**	−182.8	5.4	1.0
2	**164**	−175.6	7.1	1.8
3	**21**	−175.5	11.2	1.0
4	**242**	−169.6	1.9	1.2
5	**140**	−167.0	3.3	0.4
6	**283**	−165.4	4.8	1.7
7	**165**	−161.8	7.4	1.2
8	**235**	−161.4	5.9	0.9
9	**285**	−160.9	8.8	1.2
10	**63**	−160.1	5.2	1.1
Redocking	**MTX**	−114.2	2.2	0.3
	**DQ1**	−134.4	2.5	0.3

**Table 2:** Cinnamic acid derivatives are classified as active by combining ligand-based and structure-based VS. The numbers in italics represent those compounds classified as active in all three in silico models, but they were not previously identified as the best-ranked compounds in any approach.

Rank	Ligand	*P_LB_*_(AD)_	*P_LB_*_(VS)_	*P_SB_*	CA_Lm_
1	**63**	0.68	0.83	0.88	0.78
2	**242**	0.52	0.86	0.93	0.74
3	**96**	0.55	0.73	0.77	0.67
4	**241**	0.53	0.55	1.00	0.64
5	***39***	*0.57*	*0.64*	*0.77*	*0.64*
6	***237***	*0.61*	*0.55*	*0.84*	*0.64*
7	***306***	*0.63*	*0.53*	*0.83*	*0.63*
8	**165**	0.53	0.60	0.88	0.63
9	**140**	0.59	0.51	0.91	0.63
10	***308***	*0.57*	*0.59*	*0.81*	*0.63*

**Table 3:** Binding free energies (kJ/mol) from the MM/PBSA calculations for Compounds **237**, **306,** and **308** in the active site of *Lm*DHFR-TS; MTX was used as the reference ligand.

	237		306		308		MTX	
Energy Contribution	kJ/mol	SD	kJ/mol	SD	kJ/mol	SD	kJ/mol	SD
van der Waals	−218.3	6.2	−209.7	4.6	−217.6	6.2	−239.5	8.2
Electrostatic	−31.3	4.1	−38.0	3.9	−29.0	4.6	−57.3	4.3
Polar solvation	181.5	6.5	157.6	6.3	185.6	6.5	194.6	8.5
SASA	−23.6	1.8	−21.0	1.9	−20.0	1.2	−22.4	2.2
Binding energy	−91.6	4.7	−111.1	4.2	−81	4.6	−124.5	5.8

**Table 4:** Results of enzymatic activity against *Lm*DHFR-TS and *Hs*DHFR for selected cinnamic acid derivatives. CI = confidence interval (95%). SI = selectivity index.

Compound	*Lm*DHFR-TS		*Hs*DHFR		SI
	IC_50_ (µM)	CI (95%)	IC_50_ (µM)	CI (95%)	
hesperidin	21.6	20.2–23.1	86.5	82.3–87.2	4.0
lithospermic acid (**237**)	7.5	6.8–7.9	22.6	21.3–24.7	3.0
diarctigenin (**306**)	6.1	5.7–6.4	27.9	26.8–28.6	4.6
isolappaol A (**308**)	10.1	9.7–10.3	44.8	42.4–45.9	4.4
isovitexin 4′-*O*-glucoside	53.2	51.1–54.1	125.7	122.8–127.8	2.4
rutin	41.7	40.3–43.1	188.9	186.2–190.6	4.5
MTX	1.4	1.1–1.5	4.9	4.7–5.1	3.5

## Data Availability

The supplementary material data can be accessed directly from the authors upon request. The data are not publicly available because the data is being used in further related studies.

## Supplementary Materials

The following supporting information can be downloaded at: https://www.mdpi.com/article/10.3390/molecules29010179/s1. Table S1: Summary of the analysis results indicating the importance of molecular descriptors for classification. Table S2: Combined probabilities for the entire structure dataset obtained from random forest model and molecular docking calculations. Table S3: Prediction of pharmacokinetic properties. Table S4: Overview of Hyperparameter Optimization for Random Forest (RF) Models. Figure S1: Ramachandran plot of the *Lm*DHFR-TS. Figure S2: MLP and TPSA calculations for structures **241**, **146**, **21**, DQ1 and MTX.

## Abbreviations

3D	Three-dimensional
ADMET	Absorption, distribution, metabolism, excretion, and toxicity
APD	Applicability domain
AUC	Area under the ROC curve
BSA	Bovine serum albumin
CL	Cutaneous leishmaniasis
CYP	Cytochrome
DHFR-TS	Dihydrofolate reductase-thymidylate synthase
DNA	Deoxyribonucleic acid
E_ligand_	Ligand energy
EDTA	Ethylenediaminetetraacetic acid
E_i_	Docking energy.
E_min_	Lowest energy value
FAK	Focal adhesion kinase
*Hs*	*Homo sapiens*
HTS	High-throughput screening
IC_50_	Half-maximal inhibitory concentration
*Lm*	*Leishmania major*
*P_Lb_*	Ligand-based probability
MAPK	Mitogen-activated protein kinases
MCC	Matthew’s correlation coefficient
MD	Molecular dynamics
MLP	Molecular lipophilicity potential
MM-PBSA	Molecular mechanics Poisson–Boltzmann surface area
MTX	Methotrexate
NADPH	Nicotinamide adenine dinucleotide phosphate
NTD	Neglected tropical diseases
ns	Nanoseconds
PCA	Principal component analysis
pKa	Acid dissociation constant values
PME	Particle-mesh Ewald
PRC	Precision-recall curve
*P_SB_*	Structure-based probability
RF	Random forest
RMSD	Root-mean-square deviation
RMSF	Root-mean-square fluctuation
ROC	Receiver operating characteristic
RoG	Radius of gyration
SASA	Solvent-accessible surface area
SI	Selective index
TES	*N*-[tris(hydroxymethyl)-methyl]-2-aminoethanesulfonic acid
TNBC	Triple-negative breast cancer
TPSA	Topological polar surface area
Vo	Initial reaction rate
VS	Virtual screening
WHO	World Health Organization
