# Supplementary material for: Machine-Learning- and Structure-Based Virtual Screening for Selecting Cinnamic Acid Derivatives as Leishmania major DHFR-TS Inhibitors
Source: Molecules. 2023 Dec 28;29(1):179. doi: 10.3390/molecules29010179 (PMC10779987; doi:10.3390/molecules29010179)
Supplement: Supplementary file 1 [file molecules-29-00179-s001.zip › Supplementary_figures.pdf]

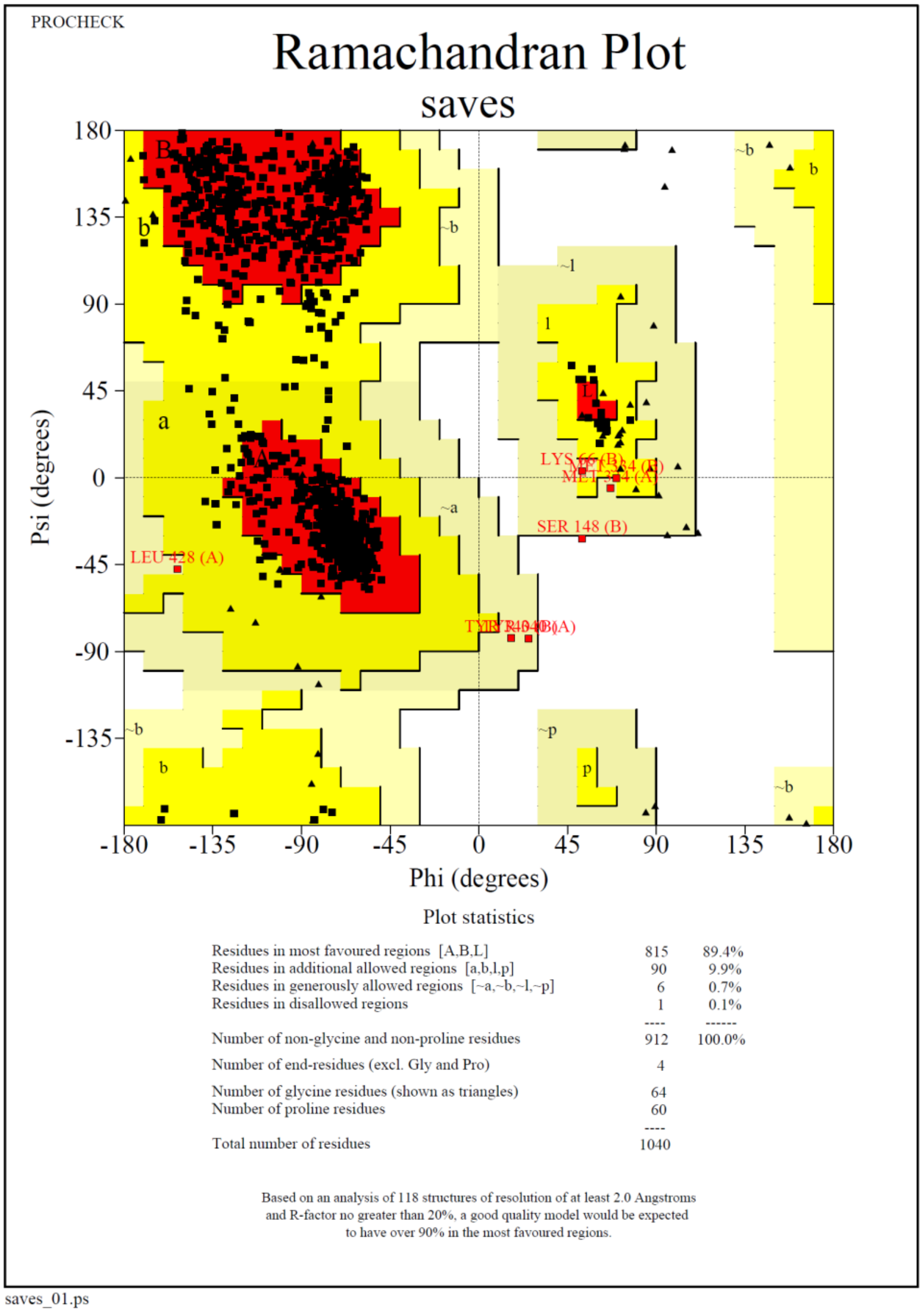

Figure S1: Ramachandran plot of the *Lm*DHFR-TS.

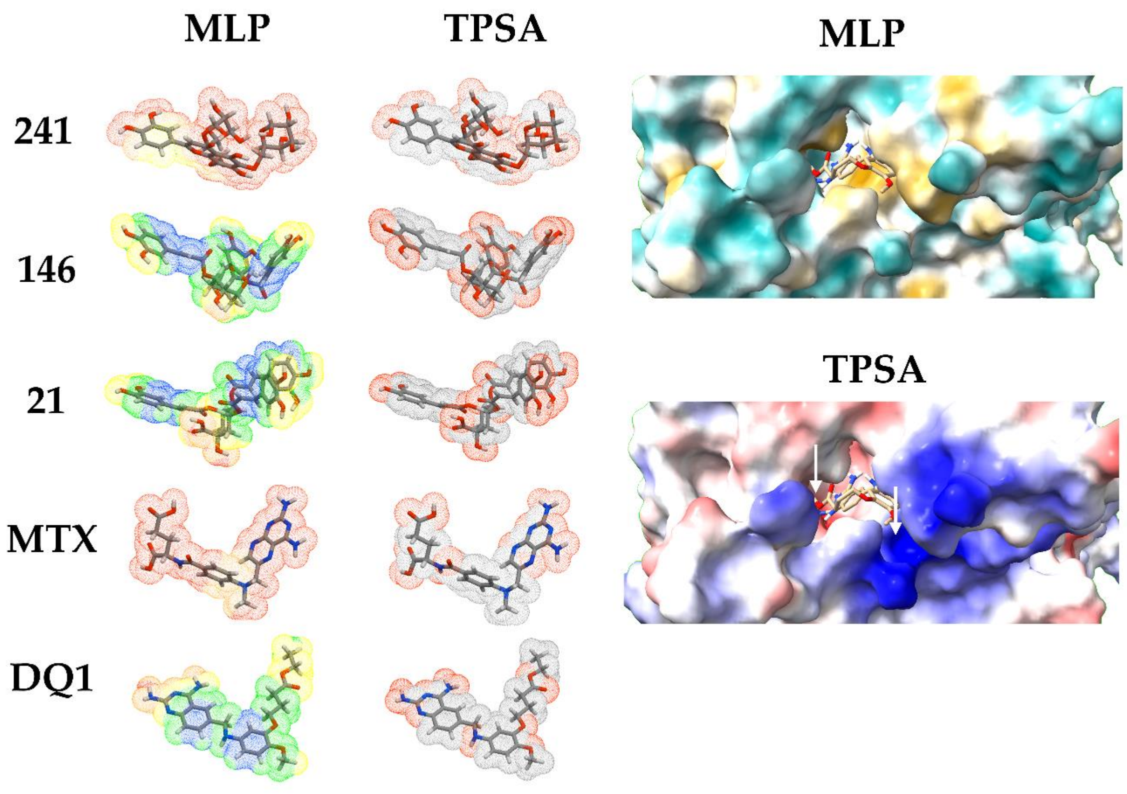

Figure S2: MLP and TPSA calculations for structures **241**, **146**, **21**, DQ1 and MTX.
